# Anthocyanins from pomegranate peel (*Punica granatum*), chili pepper fruit (*Capsicum annuum*), and *bougainvillea* flowers (*Bougainvillea spectabilis*) with multiple biofunctions: Antibacterial, antioxidant, and anticancer

**DOI:** 10.1016/j.heliyon.2024.e32222

**Published:** 2024-05-31

**Authors:** Kholoud N. Abdelrahman, Abdel Ghany A. Abdel Ghany, Refaat A. Saber, Ali Osman, Basel Sitohy, Mahmoud Sitohy

**Affiliations:** a Faculty of Development and Technology, Zagazig University, Zagazig, 44519, Egypt; b Biochemistry Department, Faculty of Agriculture, Zagazig University, Zagazig, 44519, Egypt; c Department of Clinical Microbiology, Infection, and Immunology, Umeå University, SE-90185, Umeå, Sweden; d Institution of Diagnostics and Intervention, Oncology, Umeå University, SE-90185, Umeå, Sweden

**Keywords:** Anthocyanin, *Punica granatum*, *Capsicum annuum*, *Bougainvillea spectabilis*, Phenolics, MTT-Assay, DPPH-Assay

## Abstract

**Background:**

Natural colorants, including natural pigments, e.g., anthocyanins, carotenoids, and chlorophylls, in novel and attractive food matrixes have become a popular trend. They impart favorite colors to food products and provide significant therapeutic effects. This study is aimed at extracting and identifying some natural pigments from different plant sources and evaluating their ability as antibacterial, antioxidant, and anticancer activities.

**Methods:**

The anthocyanin-rich extract (ARE) is derived from three natural plant sources: pomegranate peel (*Punica granatum*), chili pepper fruit (*Capsicum annuum*), and *Bougainvillea* flowers. *Bougainvillea spectabilis* are analyzed for biochemical composition, as well as antioxidant, antibacterial, and anticancer activity, HPLC, DPPH, FRAP, disc diffusion assay, MIC, MTT, VEGFR‐2, and caspase-9 assays.

**Results:**

All three extracts had varying total phenolic contents, ranging from 14 to 466 mg GAE/g extract, where *Punica granatum* was the highest (466 mg GAE/g extract), followed by *Bougainvillea spectabilis* (180 mg GAE/g extract), and then *Capsicum annuum* (14 mg GAE/g extract). The antioxidant activity rose steadily with raising concentration. The ARE of pomegranate peels recorded highest value, followed by *Bougainvillea* flowers and chili pepper fruit. The MTT assay revealed an inhibitory action of the tested extracts on the proliferation of HCT-116, MCF-7, and HepG2 in a concentration-based manner. Gene expression of caspase-9 transcripts was considerably multiplied by the application of ARE of pomegranate peels. All the tested extracts inhibited VEGFR-2, and the inhibition (%) expanded gradually with increasing concentrations, achieving the highest value (80 %) at 10 μg/mL. The ARE of pomegranate peels scored highest antibacterial activity, followed by ARE of chili pepper fruit and *Bougainvillea* flowers. The inhibition zone diameter escalated gradually with rising concentrations of the tested samples.

**Conclusion:**

The AREs of the three studied plant sources can be used as multifunctional products with antioxidant, anticancer, and antibacterial activities that are natural, safe, and cheap.

## 1 Introduction

Many parts of plants, including flowers, seeds, fruits, and leaves, contain anthocyanins, a class of flavonoids that are soluble in water [1], entailing pomegranate peels (*Punica granatum* L.), chili pepper fruit (*Capsicum annuum* L.), and *Bougainvillea* flowers (*Bougainvillea spectabilis* L.). Pomegranate (*Punica granatum* L.) belonging to *Lythraceae* family, has been widely investigated in recent years for its medicinal and health-care benefits, arising from a broad array of natural polyphenol compounds, including anthocyanins, flavonoids, and ellagitannin [2]. Rich color of chili pepper (Capsicum annuum) is mainly based on anthocyanins, carotenoids, and chlorophyll. Purple pepper is rich in anthocyanins and chlorophyll when fruit is mature [3]. *Bougainvillea* is a genus of colourful flowering plants within Nyctaginaceae family, containing considerable anthocyanins [4].

It has been observed that eating a diet high in phytochemicals is linked to a lower risk of long-term harm to humans, such as some forms of cancer, cardiovascular disease, and neurological disorders [[5], [6], [7], [8], [9], [10], [11]]. Therefore, biochemical evaluation of phytochemicals is paramount to highlight their potential human health benefits. Thus, a biochemical examination of them is essential to demonstrate phytochemicals' potential advantages for human health. Phenolic compounds are the primary class of antioxidant phytochemicals with advantageous characteristics because of their ability to scavenge free radicals and have biological effects. Examples of these compounds are flavonoids, anthocyanins, and tannins [7,[12], [13], [14], [15], [16], [17], [18], [19]]. Thus, anthocyanin-rich plants have become a current research objective [20]. Pomegranate has recently received particular interest for its anticancer potential. Phytomedicine is a promising choice for counteracting various forms of cancer [21]. Herbal pomegranate contains bioactive compounds, including flavonoids (mostly anthocyanin) and phenolic compounds [22]. A prominent health action of pomegranates is their potential to confront oxidative stress by reducing the number of inflammatory mediators [23]. Maintaining an appropriate physiological condition by neutralizing free radicals and antioxidants is essential to prevent and treat chronic diseases [24]. Phytochemicals such as flavanols and punicalagins, a unique class of polyphenols, are among the bioactive substances found in pomegranates. Benefits to health are attributed to flavonols and anthocyanins [25].

Impact of *Capsicum annuum* and *Capsicum frutescens* methanolic and water extracts was probed in some selected bacteria, indicating the effectiveness of both extracts against *Vibrio cholerae, Salmonella typhimurium*, and *Staphylococcus aureus*, where methanol extracts exhibited the most significant action. *Capsicum annuum* extract displayed more potent antibacterial activity than *Capsicum frutescens* extract, and an analysis of phytochemicals detected existence of polyphenols, flavonoids, alkaloids, and sterols. Therefore, capsicum fruits can serve as a natural source of antibacterial properties for both dietary and medical applications [26].

*Bougainvillea spectabilis* was reported to possess medicinal properties, viz., antidiabetic, antiviral, anti-inflammatory, antioxidant, and antifertility potentials, due to D-pinitol (3-*O*-ethylchiroinositol). Similarly, *B. glabra* had antimicrobial, antiulcer, and antidiarrheal properties. Mexican traditional medicine uses the leaves and flowers of *Bougainvillaea glabra* to treat minor respiratory tract ailments like bronchitis, coughs, and colds [27]. *Nyctaginaceae* family includes *B. spectabilis* Willd, a medicinal plant, which was first discovered in 1768 by Louis Antione de Bougainville [28]. It contains essential nutrients, including phenolic compounds, flavonoids, alkaloids, tannins, saponins, terpenoids, and mineral ions, which help fight distinct illnesses [29,30]. Researchers suggested that *B. spectabilis* possessed therapeutic benefits, including antibacterial, antioxidant, and anti-cancer. Natural products such as terpenoids, alkaloids, flavonoids, phenolics, and tannins are agents responsible for the medicinal effects. Other essential ingredients, such as bougainvinones, pinitol, quercetin, and terpinolene, support the therapeutic activities [31].

Anthocyanin fractions reduced the risk of developing intestinal cancer and inhibited the proliferation of human intestinal carcinoma HCT-15 and HCT-116 colon cancer cells [32]. The cytotoxic effects of eight distinct pomegranate cultivars against the MCF-7 and MCF-10A were estimated. The P7 cultivar exhibited highest level of cytotoxic activity against the MCF-7. The cultivars demonstrated their capacity to serve as natural antioxidants and anticancer agents [33]. According to an analysis of four different varieties of Korean green peppers, kkuri pepper, phut pepper, cheongyang pepper, and ohi pepper, Kkuri pepper emerged as the most potent antiproliferative agent. This finding suggests that the green pepper, which possesses substantial antioxidant activity, may hold promise as a therapeutic candidate for breast cancer [34].

Cancer continues to be a major cause of death worldwide, ranking second and resulting in 9.6 million fatalities in 2018 [35]. The objective of cancer therapy is to induce apoptosis in malignant cells while minimizing damage to healthy cells. Apoptosis is a controlled cellular demise mechanism that plays a crucial role in maintaining cell populations and removing impaired cells. Inducing apoptosis is a crucial approach for cancer treatment. Chemotherapy can cause cancer cell death, simulating apoptosis morphological features [36]. Nevertheless, many chemotherapeutic drugs may exert toxic effects on normal cells, particularly those dividing and growing actively, e.g., blood cells in the bone marrow. Alteration in the levels of some peptides was found to be involved in pathogenesis and treatment of colon cancer [[37], [38], [39], [40], [41], [42], [43], [44], [45]]. Plant-derived substances in natural medicines may help prevent the negative side effects associated with cancer treatment. The use of plants as medicinal remedies has a long history in treating various ailments, including cancer. Additionally, numerous studies have demonstrated that controlling plant consumption may decrease the risk of developing certain cancers [46]. Research has demonstrated the cytotoxic effects of eight stem bark peltogynoids, including *Bougainvillea* (A–H) against KB, HeLa S-3, HT-29, and HepGL. The cytotoxicity effect of compound 7 against the studied cancer cell lines recorded IC_50_ values in range of 7.4–9.7 μM while compound 2 (Bougainvinone B (C19H16O6) and compound 3 (Bougainvinone C: C_18_H_14_O_6_), demonstrated cytotoxic effects on the KB cell line, as indicated by IC_50_ values of 6.6 and 9.0 M, respectively [47].

Ultimately, the utilization of natural colorants, such as anthocyanins, carotenoids, and chlorophylls, in innovative and potentially edible formulations has gained significant traction. In addition to imparting visually appealing hues to food items, they offer consumers various beneficial properties, including antimicrobial, antioxidant, anti-inflammatory, and anticancer effects [48]. In the current study, pomegranate peel, Capsicum annuum, and Bougainvillea spectabilis as natural sources were subjected to analytical evaluation for anthocyanin content, phenolic compounds, and flavonoid contents and simultaneously were scrutinized for their antibacterial, antioxidant, and anticancer properties to discover new structure-based functionalities. Based on the previously hinted connections between antioxidant, antibacterial, and anticancer capacity, overall objective of present study was to confirm this association among the extracts of the three studied natural biosources for maximal biological impact to serve as a pioneering prephase study for using them as appropriate ingredients for healthy functional foods or specific health formulas.

## 2 Materials and methods

This investigation occurred at the Soil and Water Sciences Department Laboratory, the central lab for Soils, Foods, and Feedstuff at the Faculty of Technology and Development, and the Laboratory of Biochemistry Department, Faculty of Agriculture, Zagazig University.

### 2.1 Chemicals and reagents

DPPH (2, 2-Diphenyl-1-picrylhydrazyl), citric acid and gallic acid were purchased from Sigma Chemicals Co. (St. Louis, USA). Na2CO_3_, Folin-Ciocalteu reagent, HCl, ascorbic acid, NaOH, indophenols and organic solvents were obtained from Merck (Darmstadt, Germany).

### 2.2 Plant materials

Pomegranate peels (*Punica granatum* L. var. Wonderful), chili pepper (*Capsicum annuum* L. var., Fire Bomb), and *Bougainvillea* (*Bougainvillea spectabilis* L. var., Mrs. Butt) flowers were purchased at a Zagazig, Egypt, local market.

### 2.3 Anthocyanin-rich extract (ARE) preparation

The anthocyanin extraction from the three plant materials was carried out using acidified ethanol (85 mL ethanol, 15 mL HCl, 1.5 N) according to procedures described by Refs. [49,50] with some modifications. The fresh samples were dehydrated using liquid nitrogen and pulverized into fine powders. A quantity (40 gm) of the plant powder was placed in 400 mL of acidified ethanol and left overnight at 4 °C, before filtration on Whatman No. 1 filter paper. The remaining substance on the filter paper was then subjected to four further extractions using the same solvent. The liquid filtrates were gathered and subjected to freeze-drying.

### 2.4 Anthocyanin-rich extract characterization

#### 2.4.1 Total phenolic compounds (TPCs) determination

TPCs were estimated by Folin-Ciocalteu reagent following [51]. One mL from each sample (2000 μg/mL) was combined with 5 mL of diluted Folin-Ciocalteu reagent (1 Folin:10 distilled water, V/V) and 4 mL sodium carbonate (75 g/L) and vortex-mixed for 15 s before standing for 30 min at 40 °C. Absorbance was recorded at 765 nm. Gallic acid was utilized to prepare the standard curve at graded concentrations (10–400 μg/mL). TPCs were expressed as mg of gallic acid equivalents (GAE) per g of extract. The calibration equation for gallic acid was y = 0.0035x + 0.9212 (R^2^ = 0.9857), where y is absorbance and x is the concentration of gallic acid in μg/mL.

#### 2.4.2 Total flavonoids (TFs) determination

TFs were determined according to Ref. [52]. An aliquot (2 mL) of AlCl3 (20 g/L) ethanol solution was blended with 1 mL of the sample extract (2000 μg/mL acidified ethanol and left at 25 °C for 60 min and absorbance was recorded at 420 nm. Quercetin was applied to prepare the standard curve using graded concentrations (10–200 μg/mL). TFs were expressed as quercetin equivalent (QE) per g of extract. The calibration equation for quercetin was y = 0.0043x + 0.8437 (R^2^ = 0.9935), where y is absorbance and x is concentration of quercetin in μg/mL.

#### 2.4.3 Polyphenolic compounds and anthocyanin identification

HPLC was adopted to quantify the polyphenolic compounds in ARE crude extracts of different plant materials. The HPLC system Agilent 1100 was used as composed of a two LC- pumps pump, a UV/Vis detector, and Hypersil Gold C18column (5 μm particle size, 250 × 4.6 mm). Agilent ChemStation was used to analyze the obtained chromatograms. Two solvents, solvent A (methanol) and solvent B (acetic acid in water 1:25), made up the gradient mobile phase.

The gradient program started and lasted for the first 5 min at 100 % B, followed by 50 % eluent A for the next 10 min. The concentration of A was raised to 80 % for the subsequent 10 min and then reduced again to 50 % for the last 5 min [53]. To move the flavonoids around, a binary mixture of methanol and water (50:50 v/v) was used. The pH was set to 2.8, and the flow rate was set to 1.0 mL/min [54]. We treated and analyzed the data using the Merck Hitachi software.

For the HPLC analysis of anthocyanins in the plant extracts HPLC- LC1620A with a two LC- pumps pump, a UV/Vis detector and C18 column (125 mm × 4.60 mm, 5 μm particle size) was used. Mobile phase for the elution consisted of 0.01 % formic acid, 22.5 % HPLC-grade methanol, and 50 % HPLC-grade acetonitrile (v/v/v). Before HPLC analysis, the mobile phase and the samples underwent filtration and sonication. Flow rate was consistently maintained at 1 mL/min while measuring at a wavelength of 290 nm [55].

#### 2.4.4 FTIR (fourier-transform infrared spectroscopy)

Strctural conformation and the functional groups of ARE crude extract powders from different sources were analyzed by the FTIR technique [56]. An amount (1.0 mg) of the dried extract was added to approximately 100 mg of KBr and finely ground. The resulting powder was placed in a palletizer, forming a small thin disc, which was subsequently located in a Thermo Nicolet 380 Spectrometer (Fisher Scientific Inc., USA). The vibrational spectra were recorded at wavenumber from 4000 to 500 cm-1 and at a data acquisition rate of 2 cm^−1^ per point.

### 2.5 Antioxidant activity estimation

#### 2.5.1 DPPH-assay

Antioxidant capacity of different AREs was determined, employing DPPH assay [57]. An aliquot (500 μl) from each extract (50, 100, 200, 500, and 1000 μg/mL) was combined with 2500 μl of 0.1 mM DPPH dissolved in ethanol, incubated at 27 °C ± 3 °C for 30 min, and the absorbance was measured at 517 nm [58]. The antioxidant potential of DPPH radicals (%) was calculated using the following formula: Inhibition(%)=[(Abscontrol−Abssample)/Abscontrol]x100.

Abs control refers to the absorbance of the control, while Abs sample represents the absorbance in the presence of ARE from various sources.

#### 2.5.2 Ferric reducing antioxidant power (FRAP) method

AREs reducing power was evaluated by measuring absorption (at 700 nm) of Perls Prussian blue complex resulting from Fe^+3^ reduction into Fe^+2^, according to Ref. [59]. Ten parts of 0.3 M acetate buffer (pH 3.6), one part of 10 mM 2,4,6-tripyridyl-*s*-triazine (TPTZ) in 40 mM HCl, and one part of 20 mM FeCl3.6H_2_O in d. H_2_O were combined to create the FRAP reagent. Three mL of FRAP reagent and 0.1 mL of the extract were combined to start the reaction. The reaction proceeded for 10 min at 37 °C in the dark, and the absorbance at 593 nm was measured in comparison to a methanol-prepared blank.

### 2.6 Anticancer activity estimation

#### 2.6.1 Cell viability in vitro (MTT-assay)

The impact of AREs (crude extracts), at a concentration range of 31.25–1000 μg/mL, on human cancerous cell line viability was assessed in vitro using an MTT-assay. Normal cells (Vero cells) and cancer cells (HCT-116, MCF-7, and HepG2) were obtained from Merck (KGaA, Darmstadt, Germany). The three cell lines were cultured in DMEM medium (Sigma-Aldrich), enriched with 10 % heat-inactivated fetal bovine serum (F.B.S.), penicillin (10 U/mL, Sigma-Aldrich), and streptomycin (10 μg/mL, Sigma-Aldrich). The cultures were incubated at 37 °C, 5 % CO_2_, and 100 % relative humidity. The cells were inoculated in 96-well microplates at 10 × 10^3^ cells/well density and grown for 24 h at 37 °C in 5 % CO_2_ before adding the tested agents at various concentrations (31.25–1000 μg/mL) in phosphate-buffered saline (PBS). Cell viability was estimated after 48 h by measuring the absorbance at 550 nm [60]. A definite aliquot (10 μL of a 10 % solution) of Triton X-100 was utilized as the positive control, whereas untreated cells were used as the negative control. The following formulas calculated the percentage of cell viability and cytotoxicity:

Cellviability(%)=(Absample/Abcontrol)x100

Cytotoxic activity (%) of the tested substance was calculated following the formula:

Cytotoxicactivity(%)=100%−cellviability(%)

The ARE concentration producing 50 % growth inhibition is termed IC_50_.

#### 2.6.2 Quantification of mRNA levels of Caspase-9

The effect of ARE on apoptosis was determined by measuring the mRNA expression of Caspase-9 [61,62]. StepOnePlus real-time PCR (Applied Biosystems, Foster City, CA, USA) was employed to quantitatively analyze caspase-9 in MCF-7 and HCT-116, before and after treatments with ARE, using gene-specific primers and SYBR Green master mix. The Oligo 7 software designed the primers and then tested them for accuracy on the NCBI website. Cells were treated with IC_50_ of the ARE from different sources for 24 h. The relative expression level of caspase-9 was assessed by quantitative real-time PCR. For each sample, the average score of duplicated Ct values was measured, and the comparative Ct method was adopted to determine the target genes' relative expression levels. The primer sequences were 5ʹ-GCAGGCTCTGGATCTCGGC-3ʹ and 5ʹ-GCTGCTTGCCTGTTAGTTCGC-3ʹ for the Caspase-9 forward and Caspase-9 reverse with annealing temperature 60.5 and 59.5 ᵒC, respectively [63].

#### 2.6.3 In vitro VEGFR‐2 kinase assay

VEGFR-2 inhibitory effect of the most active antiproliferative concentrations of ARE for pomegranate peels (*Punica granatum* L.) were selected against the MCF-7 cell line, following [64]. A number of 5 × 10^5^ cells were collected per well, six wells per plate were incubated overnight in culture. ARE was added to the culture, and the medium was collected after 72 h of incubation. The levels of VEGFR-2 were assessed using a VEGF enzyme-linked immunosorbent assay (ELISA) kit (DVE00, R&D Systems, Minneapolis, MN, USA) using a specific antibody for VEGFR2 followed by the incubation with the secondary antibody following [64]. Each well's optical density was recorded at 570 nm using an automated microplate reader (model 550, BioRad, Hercules, CA, USA). Gene expression levels were compared with a control sample.

%Inhibition=[(control−treatment)/controlx100]

### 2.7 Antibacterial activity (disc assay)

Disc diffusion assay was used to evaluate the antibacterial activity of AREs from different sources against *Staphylococcus aureus, Streptococcus pyogenes, Listeria monocytogenes*, *Listeria ivanovii*) *Klebsiella oxytoca, Salmonella typhimurium, Pseudomonas aeruginosa,* and *Escherichia coli* [65], with a few modifications. The bacterial suspension was prepared by taking a loop of single colonies on Mueller-Hinton agar (MHA) plates and spreading them in 10 mL Mueller-Hinton broth (MHB), incubating at 37 °C, before reading the absorbance at 600 nm to obtain 0.5 McFarland turbidity (1.5 × 10^8^ CFU/mL). Discs (6 mm) were saturated with diverse ARE concentrations (50, 100, 250, 500, and 1000 μg/mL) and then positioned on Mueller-Hinton agar (MHA) in Petri dishes, previously inoculated with pathogenic bacterial suspensions. The MHA Petri dishes were incubated at 37 °C for 24 h. Discs saturated with sterilized distilled water were employed as the first negative control, and discs saturated with DMSO were the second negative control. A transparent ruler estimated the diameter of the inhibition zones around the discs (mm). All plates were arranged in three replicates and incubated at 28 °C for 24 h.

### 2.8 Statistical analyses

CoStat 6.311 was executed to conduct statistical analyses of the data. Duncan's test was utilized to compare the means of different treatments using a one-way ANOVA post hoc analysis. Duncan's multiple range test failed at the 5 % probability level for the means labelled with the same letter in more than one column.

## 3 Results

### 3.1 Anthocyanin-rich extract characterization

#### 3.1.1 TPCs and TFs

Table 1 presents the results of the TPC analysis of ARE (crude extract). Extraction yield (%) in three extracts ranged widely from 9.6 % to 19.5 %. The highest extraction yield was recorded in *Punica granatum* (19.5 %), while *Capsicum annuum* reflected the lowest level (9.6 %). TPCs in three extracts widely and variably ranged from 14 to 466 mg GAE/g extract. *Punica granatum* displayed remarkably the highest TPCs (466 mg GAE/g extract) followed by *Bougainvillea spectabilis* (180 mg GAE/g extract), while *Capsicum annuum* showed the lowest one (14 mg GAE/g extract). Also, the level of TFs in the three extracts varied widely from 2.62 to 228 mg QE/g extract, with the highest content appearing in Punica granatum (228 mg QE/g extract) and the lowest (2.62 mg QE/g extract) emerging in Capsicum annuum. The ARE of Punica granatum registered the highest levels of TPCs and TFs, followed by Bougainvillea spectabilis. So, the waste product pomegranate peel may be of high economic quality if adequately handled.

**Table 1:** Yield of extracted substances (%), total phenolic content (mg GAE/g extract) and total flavonoids content (mg QE/g extract) of acidified ethanol extract of Pomegranate peels (*Punica granatum* L.), Chili pepper fruit (*Capsicum annuum* L.) and Bougainvillea (*Bougainvillea spectabilis* L.) flowers.

Sample	TPCs (mg GAE/g extract)	TFs (mg QE/g extract)	Extraction yield (%)
*Punica granatum*	466 ± 0.61^a^	228 ± 0.65^a^	19.5 ± 0.64^a^
*Capsicum annuum*	14 ± 0.52^C^	2.62 ± 0.41 ^C^	9.6 ± 0.54 ^C^
*Bougainvillea spectabilis*	180 ± 0.56 ^b^	6.11 ± 0.51 ^b^	10.5 ± 0.63^b^

**TPC: Total phenolic compounds. TF: Total flavonoid compounds.** Different letters represent significant differences (Duncan's test significant difference test at p < 0.05) among all treatments. Values are showen as the mean ± SE (n = 3).

#### 3.1.2 Phenolic compounds identification

**Fig. 1:**
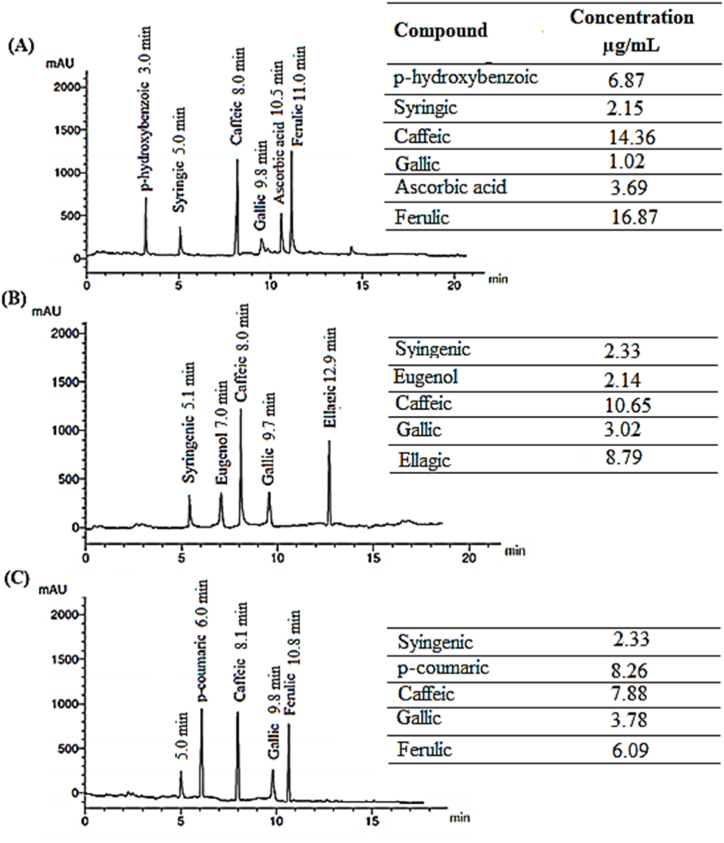
HPLC chromatogram of the major phenolic compounds in acidified ethanol extract for (A) pomegranate peels (Punica granatum L.), (B) chili pepper fruit (Capsicum annuum L.), and (C) Bougainvillea (Bougainvillea spectabilis L.) flowers.

HPLC chromatograms of the phenolic compounds of the AREs **(**crude extracts**)** of pomegranate peels, chili pepper fruit, and *Bougainvillea* flowers are illustrated in Fig. 1. The pomegranate peels exhibited 6 peaks (Fig. 1A), representing p-hydroxy benzoic, syringic acid, caffeic acid, gallic acid, ascorbic and ferulic acid. Ferulic acid and caffeic acid recorded the highest levels (16.87 and 14.36 μg/gm extract, respectively). Chili pepper (Fig. 1B) fruit manifested 5 peaks, denoting syringic acid, eugenol, caffeic, gallic, and ellagic acids, where caffeic and ellagic acids recorded the highest contents (10.65 and 8.79 μg/gm extract, respectively). Five peaks, standing for syringic, P-coumaric, caffeic, gallic, and ferulic acids, appeared in the HPLC chromatogram of Bougainvillea flowers extract (Fig. 1C), where P-coumaric and caffeic acids scored the highest contents (8.26 and 7.88 μg/gm extract, respectively).

**Fig. 2:**
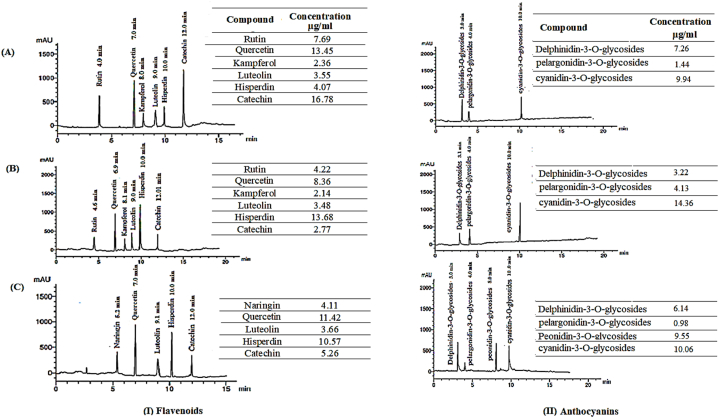
HPLC chromatogram of the major flavonoid compounds and anthocyanin in acidified ethanol extract for (A) pomegranate peels (Punica granatum L.), (B) chili pepper fruit (Capsicum annuum L.), and (C) Bougainvillea (Bougainvillea spectabilis L.) flowers.

The HPLC choromatograms of the flavonoid compounds in the ARE of pomegranate peels, chili pepper fruit, and *Bougainvillea* flowers are depicted in Fig. 2-I. Pomegranate peels (Fig. 2-I-A) produced 6 peaks (rutin, qurecetin, kampferol, luteolin, hisperdin, and catechin) where catechin and qurecetin exhibitted the highest levels (16.78 and 13.45 μg/gm extract, respectively). Chili pepper fruit (Fig. 2-I-B) pinpointed the same 6 peaks (rutin, qurecetin, kampferol, luteolin, hisperdin, and catechin) but hisperdin and qurecetin recorded highest contents (13.68 and 8.38 μg/gm extract, respectively). *Bougainvillea* flowers (Fig. 2-I-C) conveyed only 5 peaks (narigin, qurecetin, luteolin, hisperdin, and catechin(, where qurecetin and hisperdin achieved the highest concentrations (10.57 and 11.42 μg/gm extract, respectively).

#### 3.1.3 Qualitative determination of anthocyanin

HPLC chromatograms of anthocyanin in AREs for (A) pomegranate peels (*Punica granatum* L.), (B) chili pepper fruit (*Capsicum annuum* L.), and (C) *Bougainvillea* (*Bougainvillea spectabilis* L.) flowers are presented in Fig. 2 (II). For pomegranate peels (Fig. 2-II-A), three assorted glycosides were detected (Delphinidin-3-o-glucoside, Pelargonidin 3-o-glucoside, and Cyanidin-3-o-glucoside). The same pattern was observed for chili pepper fruit extract (Fig. 2-II-B). In *Bougainvillea* flowers (Fig. 2-II-C), four different glycosides were disclosed (Delphinidin-3-o-glucoside, Pelargonidin 3-o-glucoside, Peonidin-3-*O*-glucoside, and Cyanidin-3-o-glucoside), Cyanidin-3-o-glucoside recorded the highest concentrations in the three different extracts, i.e.; 9.94, 14.36 and 10.06 μg/gm, for pomegranate peels, chili pepper fruit and *Bougainvillea* flowers, respectively.

#### 3.1.4 FTIR

**Fig. 3:**
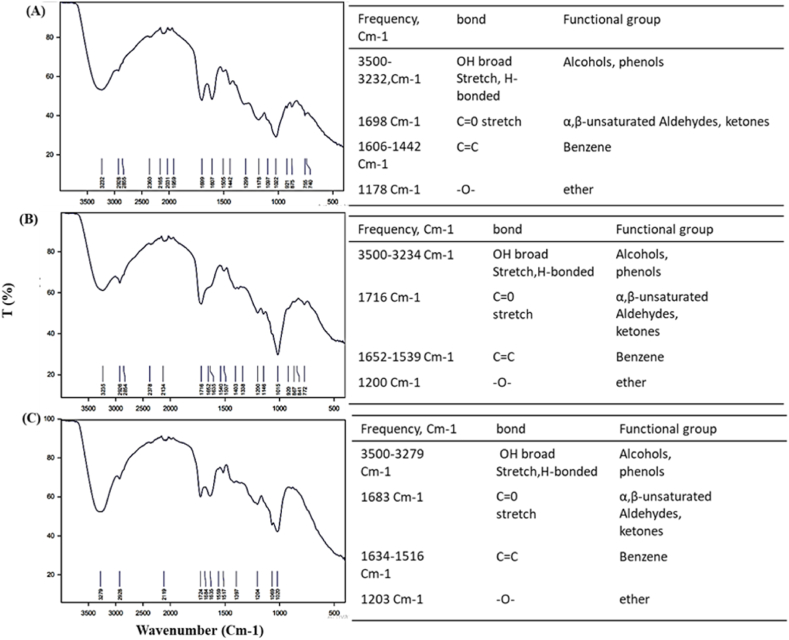
FTIR chromatogram of anthocyanin in acidified ethanol extract for (A) pomegranate peels (Punica granatum L.), (B) chili pepper fruit (Capsicum annuum L.), and (C) Bougainvillea (Bougainvillea spectabilis L.) flowers.

FTIR spectra of ARE **(**crude extract powder**)** of (A) pomegranate peels, (B) chili pepper fruit, and (C) *Bougainvillea* flowers are illuminated in Fig. 3. The infrared spectrum of pomegranate peels (Fig. 3A) expressed a peak at 3500 to 3232 cm^−1^, indicating stretching vibration bond of OH from alcohol or phenol and a peak at 1698 cm^−1^ referring to the stretching vibration bond of aliphatic C=O groups. Moreover, a peak at 1606 -1442 cm^−1^ displays the C=C group from the benzene ring, while the one at 1178 cm^−1^ marks -*O*- ether groups. The infrared spectrum of chili pepper fruit (Fig. 3B) and *Bougainvillea* flowers (Fig. 3C) unveiled similar IR spectra.

### 3.2 Antioxidant activity

#### 3.2.1 DPPH-assay

Antioxidants capacity (% inhibition) of ARE of pomegranate peels, chili pepper fruit, and *Bougainvillea* flowers at divergent concentrations is delineated in Table 2. Overall, the antioxidant activity for all tested samples grew gradually with raising concentration. ARE of pomegranate peels exposed highest value, followed by Bougainvillea flowers and then chili pepper fruit. When the concentration of pomegranate peel extract escalated from 50 to 1000 μg/mL, DPPH radical scavenging activity increased from 33 % to 82.11 %. Elevating concentration of plant extract from 50 to 1000 μg/mL intensified the DPPH radical scavenging activity of chili pepper fruit extract from 16.06 % to 25.70 and that of *Bougainvillea* flowers from 25.70 % to 58.09 %.

**Table 2:** DPPH free radical scavenging activity of acidified ethanol extract for Pomegranate peels (*Punica granatum* L.), Chili pepper fruit (*Capsicum annuum* L.) and Bougainvillea (*Bougainvillea spectabilis* L.) flowers at different concentrations.

Concentration (μg/mL)	% Inhibition		
	*Punica granatum*	*Capsicum annuum*	*Bougainvillea spectabilis*
50	33.00 ± 0.82^e^	16.06 ± 0.71 ^d^	25.70 ± 0.56^e^
100	49.11 ± 63 ^d^	18.36 ± 0.63^c^	31.24 ± 0.76 ^d^
200	60.14 ± 0.52^c^	19.47 ± 0.91^c^	36.57 ± 0.64^c^
500	70.35 ± 0.56 ^b^	22.21 ± 0.64 ^b^	47.67 ± 0.85 ^b^
1000	82.11 ± 0.42^a^	25.70 ± 0.56^a^	58.09 ± 0.45^a^

Different letters represent significant differences (Duncan's test significant difference test at p < 0.05 among all treatments). Values are showen as the mean ± SE (n = 3).

#### 3.2.2 FRAP-assay

FRAP of the three studied extracts, as indicated by optical intensity at 700 nm, is elucidated in Table 3. Altogether, the antioxidant activity for all tested samples expanded gradually with raising the extract concentration. ARE of pomegranate peels recorded the highest value (1.51–2.35 OD_700nm_), followed by Bougainvillea flowers (1.34–2.31 OD_700nm_), while chili pepper fruit exhibited the lowest values (1.07–1.35 OD_700nm_). Elevating the extract concentration from 50 to 1000 μg/mL raised the FRAP from 1.51 to 2.35 in the case of pomegranate, from 1.07 to 1.35 in the case of chili pepper fruit, and from 1.34 to 2.31 in the case of Bougainvillea flowers.

**Table 3:** Ferric reducing antioxidant power (FRAP) of acidified ethanol extract for Pomegranate peels (*Punica granatum* L.), Chili pepper fruit (*Capsicum annuum* L.) and Bougainvillea (*Bougainvillea spectabilis* L.) flowers at different concentrations.

Concentration (μg/mL)	Absorbance (OD 700 nm)		
	Punica granatum	Capsicum annuum	Bougainvillea spectabilis
50	1.51 ± 0.56^e^	1.07 ± 0.39^e^	1.34 ± 0.56^e^
100	1.65 ± 0.48 ^d^	1.24 ± 0.36 ^d^	1.47 ± 0.46 ^d^
200	1.69 ± 0.41^c^	1.26 ± 0.42^c^	1.53 ± 0.74^c^
500	1.90 ± 0.45 ^b^	1.32 ±0 .45 ^b^	1.85 ± 0.67 ^b^
1000	2.35 ± 0.32^a^	1.35 ± 0.54^a^	2.31 ± 0.69^a^

Different letters represent significant differences (Duncan's test significant difference test at p < 0.05) among all treatments. Values are showen as the mean ± SE (n = 3).

### 3.3 Anticancer activity

#### 3.3.1 MTT-assay

**Fig. 4:**
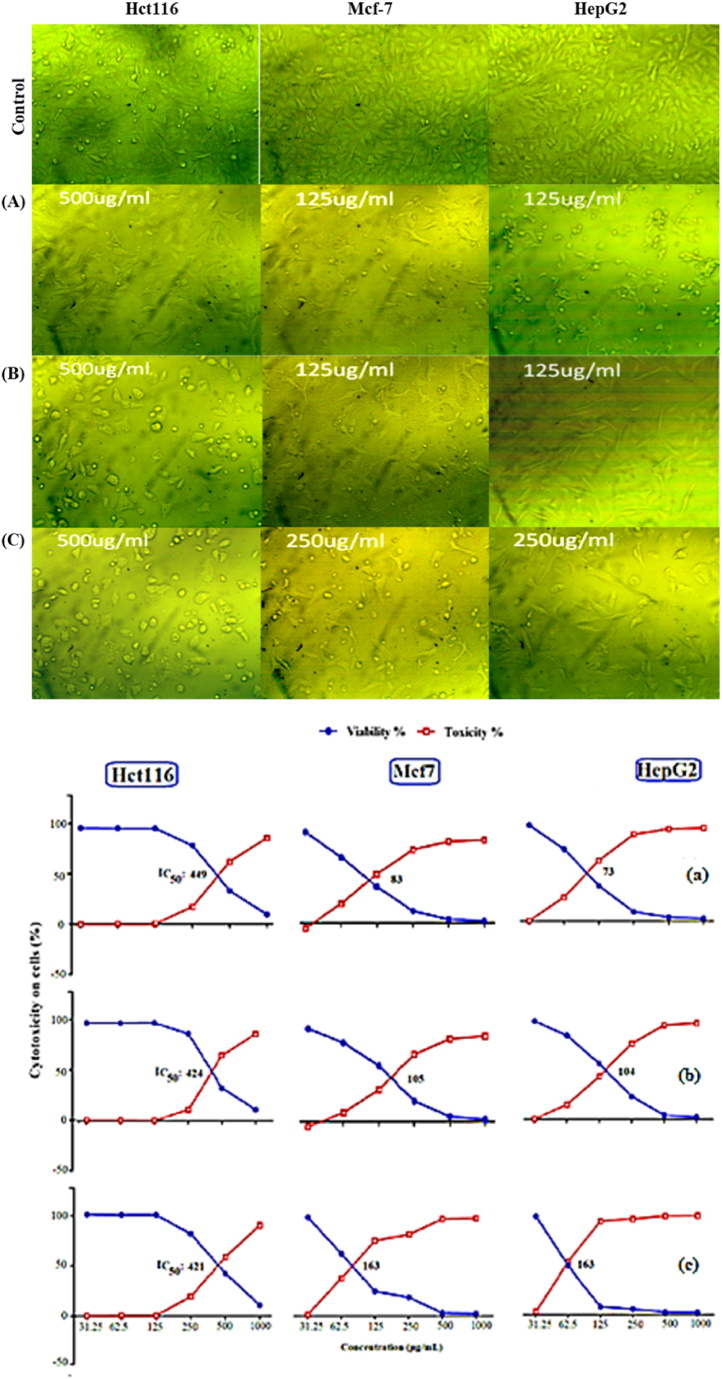
Toxicity (%) and cell viability (%) of **HCT-116**, **MCF-7**, and **HepG2** cell lines treated with acidified ethanol extract for (A) pomegranate peels (Punica granatum L.), (B) chili pepper fruit (Capsicum annuum L.), and (C) Bougainvillea (Bougainvillea spectabilis L.) flowers at opposed concentrations. The subfigures (a, b and c) represent the linear relationship between cell viability (%) and cell toxicity% under the influence of the tested substances for the three extracts, respectively.

Toxicity (%) and cell viability (%) of HCT-116, MCF-7, and HepG2 treated with AREs of pomegranate peels, chili pepper fruit, and Bougainvillea flowers at dissimilar concentrations are described in Fig. 4 (A, B and C). Subfigure 4A discloses microscopic appearance of the viable cells at the least effective concentration of the substance, while subfigure 4a represents the linear relationship between cell viability (%) and cell toxicity% under the influence of the tested substances. It is evident that the overall cell viability (%) decreased with increasing concentration of the tested samples. The MTT assay emphasized an inhibitory action of tested samples on the proliferation of HCT-116, MCF-7, and HepG2 in a concentration-based manner. In the ARE of pomegranate peels (Fig. 4a), the lowest IC_50_ was marked against HepG2 (73 μg/mL), followed by MCF-7 (83 μg/mL) while HCT-116 registered relatively higher value (449 μg/mL). For chili pepper fruit (Fig. 4b), the lowest IC_50_ was noticed against HepG2 (104 μg/mL), followed by MCF-7 (105 μg/mL), while HCT-116 was associated with a relatively bigger value (424 μg/mL). For *Bougainvillea* flowers (Fig. 4c), the lowest IC_50_ was reported against HepG2 and MCF-7 (163 μg/mL), followed by HCT-116 (421 μg/mL).

**Fig. 5:**
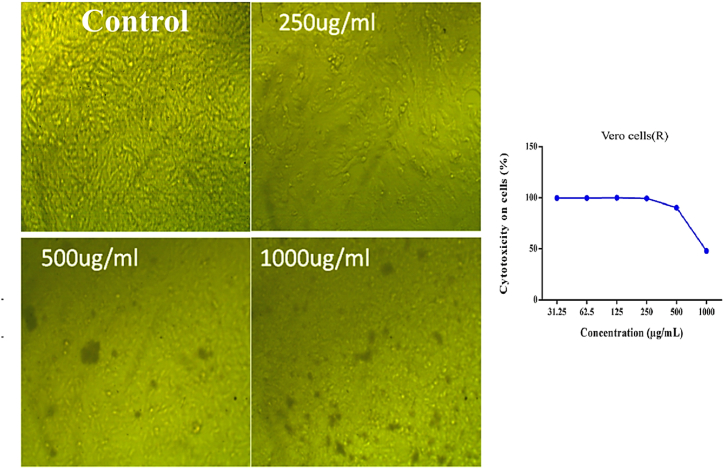
Potential toxicity of pomegranate peels (Punica granatum L.) ARE on **Vero** cell line.

Normal cells (Vero cells) were treated with pomegranate ARE at a concentration range of 31.25–1000 μg/mL. The low concentrations of pomegranate ARE (31.25, 62.5, and 125 μg/mL) did not produce any toxicity in Vero cell. Only 10 % toxicity was observed in Vero cells at the medium concentration of 500 μg/mL, while 52 % toxicity was noticed at 1000 μg/mL of pomegranate ARE (Fig. 5).

#### 3.3.2 Quantification of mRNA levels of Caspase-9

**Fig. 6:**
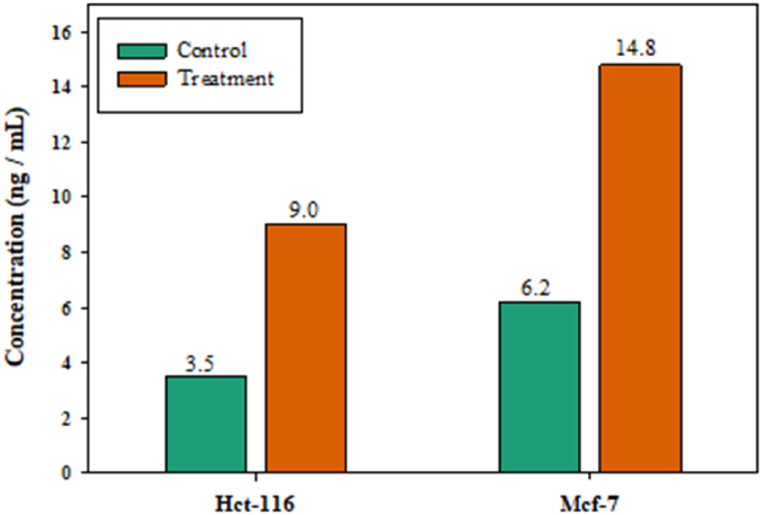
The effect of acidified ethanol extract from pomegranate peels (Punica granatum L.) on caspase-9 mRNA expression of two human cancer cell lines (**HCT-116**-116 and **MCF-7**). Cells were treated with the concentration causing the IC_50_ in each cell line for 24 h and their mRNA levels were evaluated by quantitative real-time PCR.

Exploring the molecular mechanism of apoptosis in HCT-116 and MCF-7 induced by ARE of pomegranate peels necessitated estimating caspase-9 expression (Fig. 6). Gene expression of caspase-9 transcripts seems highly multiplied by the application of ARE of pomegranate peels. Treating HCT-116 and MCF-7 with IC_50_ (449 and 83 μg/mL) ARE of pomegranate peels for 24 h enhanced the expression of caspase-9 by 2.6 and 2.4 folds, respectively, compared to the levels of the untreated control.

#### *3.3.3* *In vitro* VEGFR‐2 kinase

**Fig. 7:**
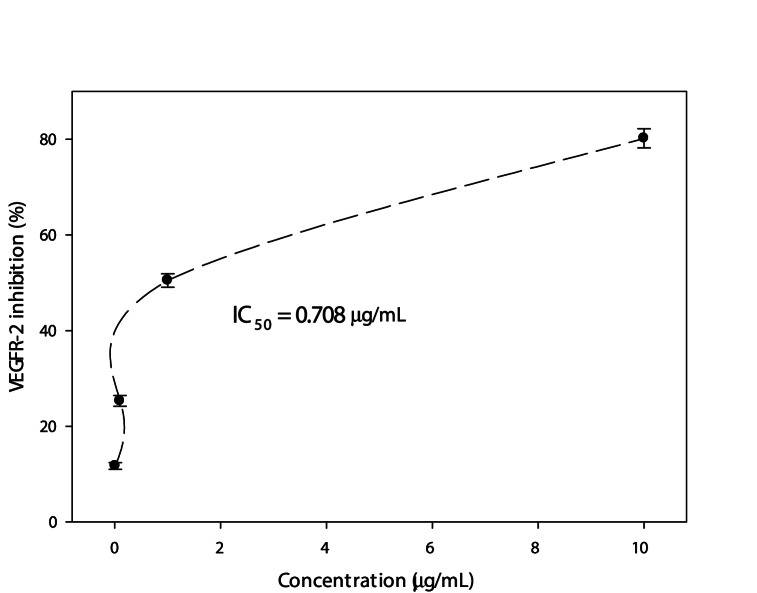
The inhibition (%) of VEGFR-2 in **MCF-7** cell line after treatment with acidified ethanol extract from pomegranate peels (Punica granatum L.) at contrasting concentrations.

The inhibitory action of ARE of pomegranate peels against the biomarker VEGFR‐2 in the cell line MCF-7 was evaluated at opposed concentrations of the extract, using an anti-phosphoserine antibody within the PerkinElmer AlphaScreen system; the results are depicted in Fig. 7. VEGFR-2 inhibition (%) was reinforced gradually with increasing the extract concentration. The highest value (80 %) was remarked at 10 μg/mL, while the IC_50_ was concluded at 0.708 μg/mL of ARE of pomegranate peels.

### 3.4 Antibacterial activity

The antibacterial activities estimated as the inhibition zones induced by the three studied AREs of plant sources at opposed concentrations were assessed against studied bacteria, and the results are shown in Table 4. The highest antibacterial activity was triggered by the ARE of pomegranate peels, followed by chili pepper fruit and Bougainvillea flowers, at all the tested extract concentrations. The inhibition zone diameter rose gradually by boosting the extract concentrations of all tested samples. The MIC. values of the contrasting extracts are documented in Table 5. The ARE of pomegranate peels registered the lowest MIC (1 μg/mL) against both Gram-positive and Gram-negative bacteria, while the other two extracts had relatively higher MIC (5 μg/mL) against both Gram-positive and Gram-negative bacteria, respectively. Minimum inhibitory concentration (MIC) was estimated using conventional broth dilution assay [66].

**Table 4:** Antibacterial activity of acidified ethanol extracts of pomegranate peels (*Punica granatum* L.), Chili pepper fruit and Bougainvillea flowers against gram-positive and gram-negative bacteria at different concentrations.

		Concentration (μg/mL)				
		50	100	250	500	1000
		Inhibition zone (mm) after24hrs.				
Microorganisms		**Pomegranate peels acidified ethanolic extract**				
G+	*Staph. aureus*	8.13 ± 0.54^e^	9.22 ± 0.47^h^	10.23 ± 0.91^h^	12.98 ± 0.54^h^	17.87 ± 0.43^e^
	*Strept. pyogenes*	8.44 ± 0.54^b^	9.33 ± 0.65^g^	10.55 ± 0.84^e^	14.14 ± 0.65^d^	17.11 ± 0.65^g^
	*L. monocytogenes*	8.12 ± 0.43^f^	9.95 ± 0.43^a^	10.77 ± 0.49^c^	13.69 ± 0.72^g^	16.34 ± 0.74^h^
	*L. ivvanovi*	8.43 ± 0.54^c^	9.66 ± 0.65^c^	10.61 ± 0.84^d^	13.98 ± 0.54^f^	17.79 ± 0.65^f^
G-	*K. oxytoca*	7.49 ± 0.42^h^	9.87 ± 0.91b	10.89 ± 0.42^a^	14.11 ± 0.65^e^	18.11 ±0 .92^c^
	*S. typhimurum*	7.77 ± 0.58^g^	9.44 ± 0.75e	10.42 ± 0.61^f^	15.55 ± 0.45^b^	18.12 ± 0.74^b^
	*P. aeruginosa*	8.66 ± 0.65^a^	9.54 ± 0.62d	10.38 ± 0.54^g^	15.66 ± 0.64^a^	17.89 ± 0.43^d^
	*Escherichia coli*	8.38 ± 0.56^d^	9.34 ± 0.54f	10.88 ±0 .62^b^	15.11 ±0 .62^c^	18.67 ± 0.51^a^
**Chili pepper fruit acidified ethanol extract**						
G+	*Staph. aureus*	7.10 ± 0.54^h^	7.16 ± 0.54 ^h^	8.44 ± 0.85^c^	10.35 ± 0.25^e^	11.77 ± 0.54^g^
	*Strept. pyogenes*	7.12 ± 0.58^g^	7.62 ± 0.65^d^	8.35 ± 0.23^e^	10.51 ± 0.45^c^	12.22 ± 0.85^e^
	*L. monocytogenes*	7.13 ± 0.62^f^	7.43 ± 0.74^f^	8.59 ± 0.84^b^	10.65 ± 0.93^b^	11.44 ± 0.65^h^
	*L. ivvanovi*	7.17 ± 0.45^e^	7.37 ± 0.61^g^	8.31 ± 0.65^g^	10.43 ± 0.52^d^	12.29 ± 0.25^c^
G-	*K. oxytoca*	7.32 ± 0.65^a^	7.52 ± 0.75^e^	8.82 ± 0.25^a^	10.23 ± 0.25^f^	12.32 ± 0.48_b_
	*S. typhimurum*	7.22 ± 0.54^c^	7.77 ± 0.62^b^	8.31 ± 0.48^g^	10.14 ± 0.48^g^	12.88 ± 0.45^a^
	*P. aeruginosa*	7.23 ± 0.87^b^	7.73 ± 0.84^c^	8.33 ± 0.94^f^	10.83 ± 0.32^a^	12.24 ± 0.81^d^
	*Escherichia coli*	7.21 ± 0.65^d^	7.84 ± 0.43^a^	8.37 ± 0.84^d^	10.11 ± 0.81^h^	11.99 ± 0.54^f^
**Bougainvillea flowers acidified ethanol extract**						
G+	*Staph. aureus*	6.03 ± 0.36^c^	7.13 ± 0.51^g^	7.97 ± 0.32^g^	8.49 ± 0.14^f^	10.56 ± 0.21^f^
	*Strept. pyogenes*	6.00 ± 0.48^f^	7.10 ± 0.05^h^	7.88 ± 0.23^e^	8.38 ± 0.32^h^	10.48 ± 0.32^g^
	*L. monocytogenes*	6.04 ± 0.41^b^	7.14 ± 0.52^f^	7.86 ± 0.19^f^	8.79 ± 0.54^e^	10.85 ±0 .09 ^b^
	*L. ivvanovi*	6.01 ± 0.87^e^	7.15 ± 0.09^e^	7.91 ± 0.09^c^	8.39 ± 0.32^g^	10.57 ± 0.45^e^
G-	*K. oxytoca*	6.05 ± 0.31^a^	7.19 ± 025^c^	7.98 ± 0.58^a^	8.82 ± 0.58^d^	10.63 ± 0.32^d^
	*S. typhimurum*	6.04 ± 0.07^b^	7.20 ± 00.61^b^	7.78 ± 0.41^h^	8.92 ± 0.25^c^	10.66 ± 0.16^c^
	*P. aeruginosa*	6.02 ± 0.19^d^	7.18 ± 0.42^d^	7.93 ± 0.24^b^	8.94 ± 0.09^b^	10.88 ± 0.48^a^
	*Escherichia coli*	6.05 ± 0.24^a^	7.21 ± 0.64^a^	7.89 ± 0.48^d^	8.95 ± 0.48^a^	10.44 ± 0.48^h^

G^+^: (Gram-positive bacteria). G^−^: Gram-negative bacteria (−). Different letters represent significant differences (Duncan's test significant difference test at p < 0.05) among all treatments. Values are showen as the mean ± SE (n = 3).

**Table 5:** Minimum inhibitory concentration (MIC) of acidified ethanol extract for Pomegranate peels (*Punica granatum* L.), Chili pepper fruit (*Capsicum annuum* L.) and Bougainvillea (*Bougainvillea spectabilis* L.) flowers against gram-positive and gram-negative bacteria.

Microorganisms		MIC (μg/mL)		
		*Punica granatum*	Capsicum annuum	Bougainvillea spectabilis
**G (+)**^**1**^	*Staphylococcus aureus*	1	5	5
	*Streptococcus pyogenes*	1	5	5
	*Listeria monocytogenes*	1	5	5
	*Listeria ivvanovi*	1	5	5
**G (−)**^**2**^	*Klebsiella oxytoca*	1	5	5
	*Salmonella typhimurum*	1	5	5
	*Pseudomonas aeruginosa*	1	5	5
	*Escherichia coli*	1	5	5

## 4 Discussion

Natural pigments (e.g., anthocyanins) are gaining accelerating significance in food industries because of their nontoxic and ecofriendly characteristics [67], as well as their abundancy and renewability and their versatile biological activities, e.g., antibacterial, antioxidant, and anticancer agents [68]. In the current study, pomegranate peel, *Capsicum annuum*, and *Bougainvillea spectabilis* as natural sources were subjected to evaluation for anthocyanin content, flavonoid contents, and antibacterial, antioxidant, and anticancer properties. Phenolic compounds exert anticancer effects by affecting proliferation, developing apoptosis, and inhibiting angiogenesis in agreement with [69]. This anticancer is due to TPCs, TFs and anthocyanin content as well as their radical scavenging activity in accordance with [70].

In the present study, ARE of *Punica granatum* showed remarkably high total phenolic content in accordance with [71], followed by *Bougainvillea spectabilis,* while *Capsicum annuum* expressed the lowest content, and the same pattern was documented for total flavonoids. The data highlighted a correlation between total antioxidant capacities obtained from DPPH and FRAP and TPCs of three extracts. The results demonstrated a favorable linear relationship between the overall antioxidant activities and the total phenolic contents, as stated in Ref. [72]. The TPCs significantly contributed to the antioxidant activity of the plant species, as demonstrated by these results. Additionally, other authors have documented the correlation between the antioxidant potential of the extract and its TFC content [73,74]. The effectiveness of anthocyanins as antioxidants relies on their fundamental structural orientation, specifically the arrangement of their rings. This arrangement determines the accessibility of H^+^ from OH^−^ to be given to a free radical, while also providing support for an unpaired electron [75]. The particularly higher antioxidant capacity of ARE of *Punica granatum* than the other two plant sources is based on its higher contents of TPCs and TFs contents and agrees with the results of [76].

The results of the present study noted an anticancer activity against the HCT-116, MCF-7, and HepG2. MTT assay revealed an inhibitory action of the tested samples on the proliferation of HCT-116, MCF-7, and HepG2 in a concentration-based manner. These data suggest that anticancer potential of ARE for pomegranate peels (*Punica granatum* L.), chili pepper fruit (*Capsicum annuum* L.), and *Bougainvillea* (*Bougainvillea spectabilis* L.) flowers may be associated with high content of phenolic compounds in this species in accordance with [77,78]. Research has documented the anticarcinogenic properties of anthocyanins against various cancer cell lines [79].The IC_50_ of ARE of pomegranate peels marked low values against the cancerous cell lines, i.e., 73, 83, and 449 μg/mL against **HepG2**, **MCF-7,** and **HCT-116**, respectively, unlike a relatively a high number (1000 μg/mL) on the normal Vero cells. This adds to the distinct effectiveness and safety of ARE of pomegranate peels. Consequently, the antibacterial action of ARE was concentration dependent. The strong antibacterial action of ARE of pomegranate peels agrees with the results of [80] and is linked to its high content of phenolic compounds agreeing with [81].

The observed highly multiplied gene expression of caspase-9 by the application of ARE of pomegranate peels on HCT-116 and MCF-7 cells (2.4–2.6 folds) may partially explain its molecular mechanism. Caspase-9 is a key player involved in various stimuli and should be activated via a plenty of intrinsic proteins and small molecules to maintain it catalytic status [82]. Hence, the biological activity of the manifold phenolic compounds registered in ARE, particularly anthocyanins, may have some activation action on caspase-9 and consequently on the apoptotic action. Antiapoptotic activity of anthocyanins was detected to have a potential to inhibit Caspase-3 signaling [83,84].

The remarkable inhibitory action of the ARE of pomegranate peels against the biomarker VEGFR‐2 in the cell line MCF-7, which reached 80 % at 10 μg/mL and achieved IC50 at 0.708 μg/mL confirms it as an anticancer, probably suggesting a different mode of action since VEGFR-2 is the most significant transducer of the vascular endothelial growth factor- (VEGF-)dependent angiogenesis [85]. Moreover, inhibiting VEGFR-2 was found to be efficient in the early stages of angiogenesis [86]. In accordance with Teller et al., working on anthocyanin-rich extract of bilberries and grapes, this action may result from the phenolic compounds in this extract, particularly anthocyanins [87]. Inhibiting the VEGF/VEGFR signaling pathway is a promising therapeutic target for controlling tumor angiogenesis and preventing potentially subsequent tumor growth [88]. Consequently, ARE of pomegranate peels can be a good candidate for counteracting cancer development.

The results confirmed high antibacterial activity of ARE of pomegranate peels, which registered the lowest MIC (1 μg/mL) against G+ and G-bacteria, while other two extracts reflected relatively higher MIC (5 μg/mL) against both bacterial types. This comes in accordance with a research team [89] who studied the antimicrobial activities of eight food dyes against ten bacteria and five fungi, observing the association between the red dyes with the best antibacterial activities. The red dye, e.g., anthocyanins, was also found to have greater antioxidant activity in all methods. This broad-spectrum antibacterial activity of these products agrees with other natural products [[90], [91], [92], [93], [94], [95]], since the modes of action of most of them are similar, targeting the bacterial membranes. However, the level of antimicrobial activity in this group is much higher than the other natural products based on very low levels of MIC (1–5 5 μg/mL) compared to levels more than 50 μg/mL in other natural products [[96], [97], [98]]. The association between the antioxidant and antibacterial activities in these ARE products may be similar to other natural products such as phycocyanins [99,100]. This antibacterial activity is very important for the anticancer agent since an association was reported between cancer development and bacterial infection [101,102].

## 5 Conclusions

The ARE from the natural sources, pomegranate peel, *Capsicum annuum* fruits, and *Bougainvillea spectabilis* flowers, exhibited high levels of anthocyanin content, flavonoid contents, and conveyed multiple and high biological activities, especially as antioxidant and anticancer factors. The ARE of pomegranate peel was the highest in phenolic compounds contents and anticarcinogenic and antibacterial activities. Antioxidant capacity was a result of the high levels of phenolic compound and the anticarcinogenic actions were further proved by the distinguished multiplication action of ARE of pomegranate peel on caspase-9 expression and the inhibitory action on the biomarker VEGFR‐2 in the cell line MCF-7. The combination of the three activities, antioxidant, anticancer, and antibacterial, in one product may nominate these extracts as potentially effective food additives for healthy foods and as natural health-protective agents. Potential use of ARE of pomegranate as an anticancer agent may have the advantage to counteract any possible bacterial contamination in the cancer patients. These products can be effectively and successfully used as safe, natural products and can be prepared at low costs. Utilization of natural bioresources, such as pomegranate peel (an inexpensive waste product), to produce ARE holds great potential for manifold applications in the development of high-quality, nutritious, and functional foods. These extracts have shown promise in providing protection against cancer, combating aging processes, and fighting against microbial infectious diseases. This conclusion is based on the evidenced antibacterial, antioxidant, and anticancer activities of these natural ARE products, asserting the connection between these three types of activities. However, further studies may be needed to define and precisely explain the mechanisms governing each activity and their coordination. Animal experimentation is a must-have in the future to prove the interactions, potential synergy, or coordination inside the living organism. Likewise, proving the potential applications of these areas, either as functional foods or drugs, may need further detailed studies. Fractionation of the extract components may help delineate a more precise relationship between the impacting factor and the produced biological effect.

## Funding

This study was funded by 10.13039/501100007102Zagazig University, Zagazig, Egypt, and the County Council of Västerbotten (BS), Lions Cancer Research Fund (BS), and 10.13039/501100007067Kempestiftelserna (BS), Sweden.

## Data availability statement

The datasets used and analyzed during the current study are available from the corresponding author upon reasonable request.

## Additional information

No additional information is available in this paper.

## CRediT authorship contribution statement

**Kholoud N. Abdelrahman:** Methodology, Formal analysis. **Abdel Ghany A. Abdel Ghany:** Supervision, Investigation. **Refaat A. Saber:** Supervision, Investigation. **Ali Osman:** Writing – review & editing, Methodology, Formal analysis. **Basel Sitohy:** Writing – review & editing, Writing – original draft, Visualization, Conceptualization. **Mahmoud Sitohy:** Writing – review & editing, Writing – original draft, Validation, Conceptualization.

## Declaration of competing interest

All authors declare that there is no conflict of interest in this research.
