# Supplementary figures and images for: Anthocyanins from pomegranate peel (Punica granatum), chili pepper fruit (Capsicum annuum), and bougainvillea flowers (Bougainvillea spectabilis) with multiple biofunctions: Antibacterial, antioxidant, and anticancer
Source: Heliyon. 2024 May 31;10(11):e32222. doi: 10.1016/j.heliyon.2024.e32222 (PMC11168436; doi:10.1016/j.heliyon.2024.e32222)

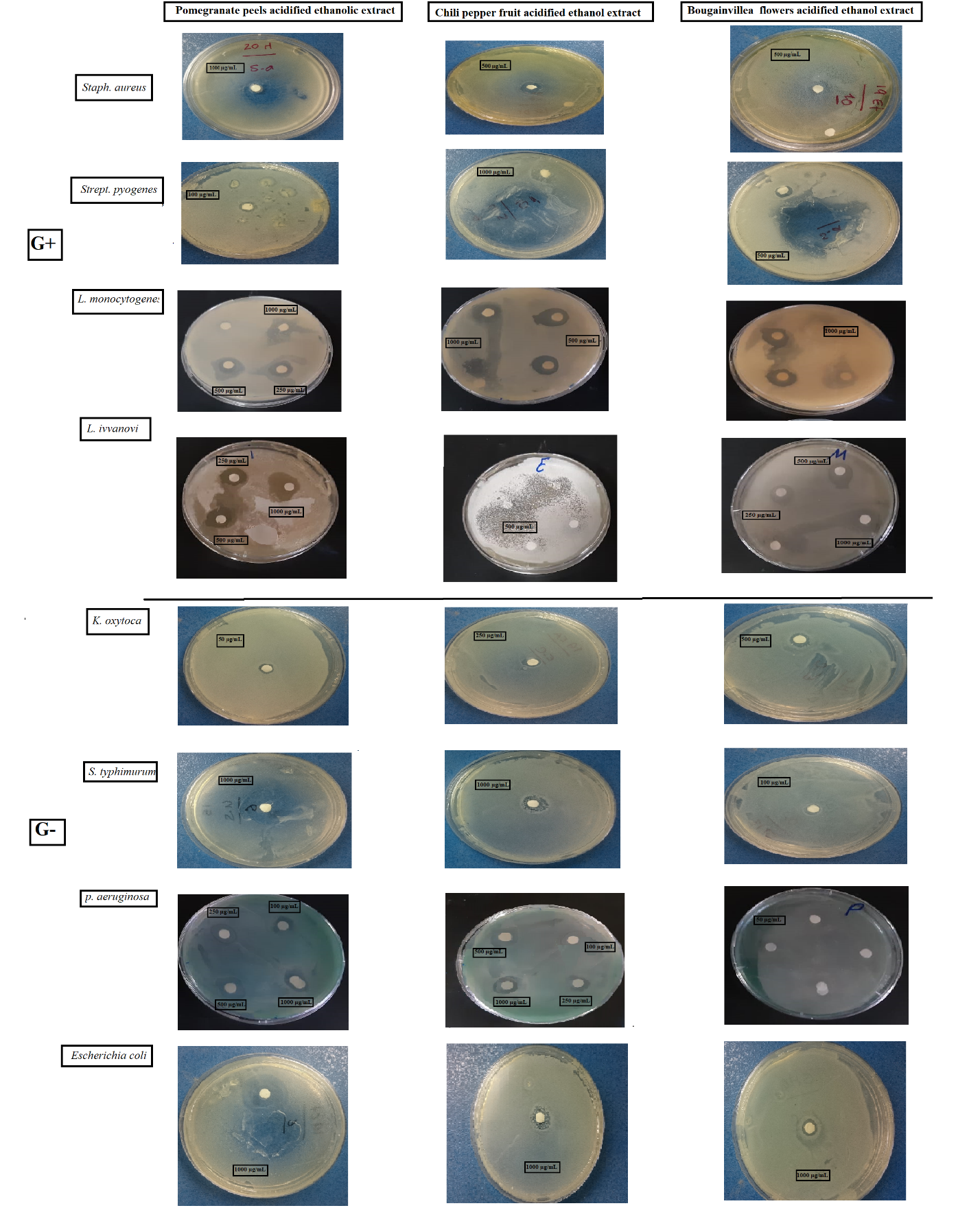


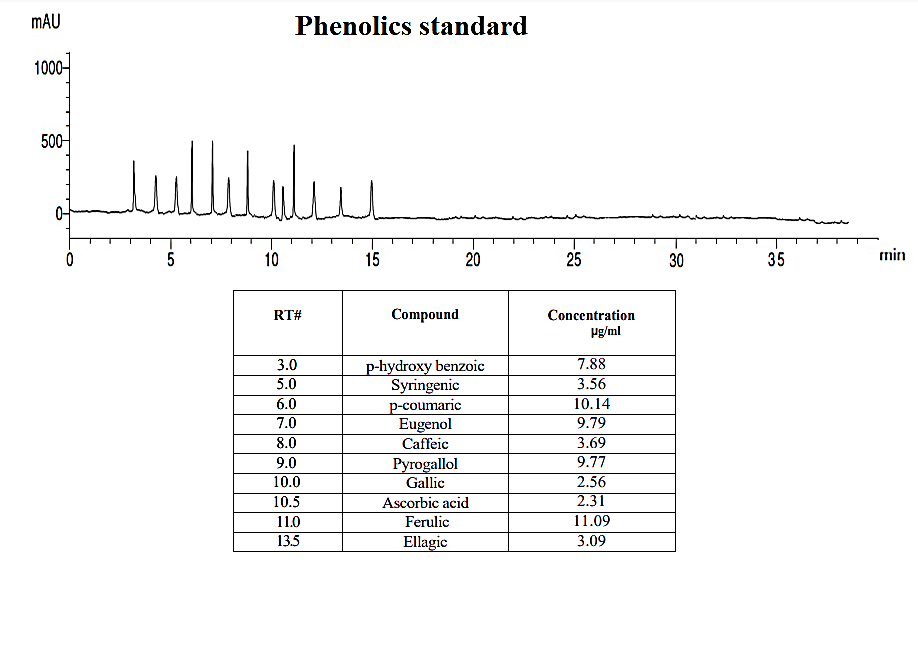


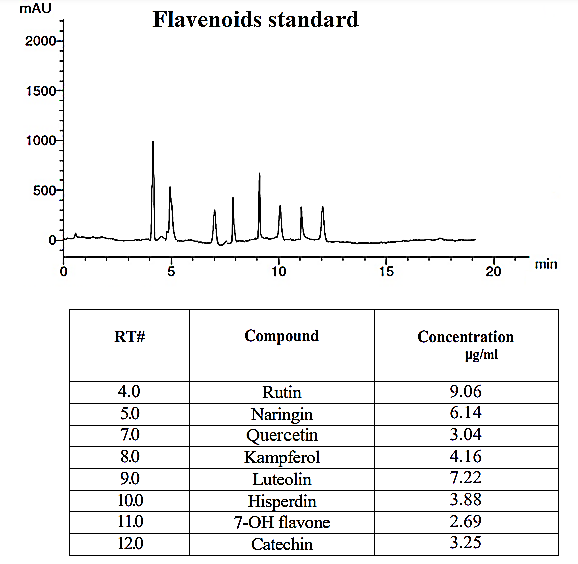

Supplement: Multimedia component 1 [file mmc1.docx]
